# Comparative Analysis of Preoperative Templating in Total Hip Replacement Surgery: KingMark™ Dual-Marker System Versus Single-Marker Method

**DOI:** 10.7759/cureus.83501

**Published:** 2025-05-05

**Authors:** Annis Maatough, Hany Elbardesy, Mohammad Mirza, Ali Hussain, Nicolas Atte, Suresh Kondi, Ketan Kantamaneni, Nimesh Patel, Tofunmi Oni

**Affiliations:** 1 Trauma and Orthopaedics, East Kent Hospitals NHS Trust, East Kent Hospitals University NHS Foundation Trust, Ashford, GBR; 2 Trauma and Orthopaedic Surgery, Cork University Hospital, Cork, IRL; 3 Orthopaedics, East Kent Hospitals NHS Trust, East Kent Hospitals University NHS Foundation Trust, Ashford, GBR; 4 Trauma and Orthopaedics, Norfolk and Norwich University Hospitals NHS Foundation Trust, Cambridge, GBR; 5 Trauma and Orthopaedics, Queen Alexandra Hospital, Portsmouth, GBR; 6 Trauma and Orthopaedics, East Kent Hospitals NHS Trust, East Kent Hospitals University NHS Foundation Trust, Margate, GBR

**Keywords:** acetabular component, femoral component, hip arthroplasty, implant size prediction, kingmark™ dual-marker system, preoperative templating, radiographic calibration, single-marker method, total hip replacement (thr), x-ray magnification factor (xmf)

## Abstract

Purpose of the study

Total hip replacements (THRs) are a standard and effective surgical procedure that benefits from preoperative planning. Despite this, no consensus exists on the best preoperative templating tool for THRs. In this study, we compare the single marker to the KingMark™ double-templating system for predicting the size of implants used intraoperatively.

Methods

This retrospective study compares two cohorts of 50 consecutively selected patients who underwent primary THR under the care of two orthopaedic surgeons. All patients had preoperative anteroposterior (AP) pelvic radiographs to facilitate templating by one of the two methods. The first cohort had surgery with single-marker templated THRs from August to December 2021. The second cohort had THRs templated using the KingMark™ system and underwent surgery between January and April 2022. For both groups, the templated size of the acetabular and femoral implants was compared to the definitive acetabular and femoral implants, respectively. Any patients with a history of previous hip surgery, with developmental abnormality affecting hip anatomy, or requiring bespoke implants were excluded.

Results

Single-marker templating accurately predicted the femoral implant size in 32% of cases. KingMark™ correctly predicted femoral implant size in 54% of cases, a statistically significant improvement (p=0.04). The mean templated acetabular cup size for the single-marker cohort templated acetabular size was 52.5±4.1, and the definitive acetabular size was 53.6±3.5. The mean templated acetabular cup size for the KingMark™ cohort was 52.0±3.7, and the definitive acetabular cup size was 53.2±4.8. The absolute difference between templated and definitive acetabular implants was 2.3±2.4 and 2.2±2.6, respectively, which was not statistically significant (p=0.84). This is consistent with the rate of accurate acetabular implant prediction for both templating methods (32% for single marker and 30% for KingMark™) with no significant difference (p=0.83).

Conclusion

The KingMark™ system showed superior accuracy in predicting the femoral stem size in THR over the conventional single mark. However, it's important to note that there was no significant difference between the two methods in predicting the cup size, a key finding of our study.

## Introduction

Total hip replacement (THR) is one of the most frequently performed surgical procedures in the United Kingdom and is amongst the most effective, with a 97% success rate in the National Health Service (NHS) [1]. Preoperative templating of THRs provides surgeons with a more accurate intraoperative plan with respect to implant size and positioning [2,3]. Furthermore, it has been shown to improve leg length restoration and reduce intraoperative complications [4]. The widespread utilisation of digital imaging in picture archiving and communication systems (PACS) has led to the increasing use of computer-automated design (CAD) for surgical templating. CAD software results in more reliable and reproducible templating; however, this relies on the software accurately calibrating input images' X-ray magnification factor (XMF). The accuracy of preoperative templating can be improved with more accurate calibrating tools to determine the XMF [4,5]. The standard method of determining the XMF by placing a marker of known size within the radiograph has satisfactory reported outcomes [6-9].

Single-marker calibration requires placing a marker between the thighs or at the greater trochanter level; the CAD software uses this as a size scale to calibrate the XMF [5,10]. The single-marker method depends on the distance of the marker from the X-ray detector, and for accurate templating, this must be the same as the distance of the femoral head to the detector [10,11]. Brainlab's single-marker system (VoyantMark™) has an adjustable arm for the marker to be placed at the patient's greater trochanter level. Accurate placement depends on the radiographer's ability and the patient's body habitus [12]. Inconsistency in marker placement leads to the inaccurate calibration of XMF and preoperative templating.

Brainlab's dual-marker system (KingMark™) involves standardised radiopaque objects placed anterior and posterior to the patient as an alternative method of determining XMF [11]. This method does not rely on estimating the location of the patient's femoral head and has been reported to be four times more accurate than the single-marker method [11,13,14]. Other studies have demonstrated no significant difference in predicting implant sizes for THRs [15].

Understanding the differences between these two methods is crucial for clinical practice and implant planning. Accurate prediction of implant size is vital not only for the immediate success of THRs but also for the long-term functionality and longevity of the implant. Improved accuracy in sizing could lead to better patient outcomes, reducing the risk of complications such as implant loosening or instability. By evaluating the effectiveness of the KingMark™ method against the traditional single-marker approach, this study seeks to provide the most suitable method for predicting definitive implant size in THRs.

## Materials and methods

Study design and patient selection

This retrospective study evaluates two cohorts of 50 consecutively selected patients undergoing primary THR conducted by two orthopaedic surgeons at Kent and Canterbury Hospital (Canterbury), William Harvey Hospital (Ashford), and Queen Elizabeth The Queen Mother Hospital (Margate), East Kent Hospitals University NHS Foundation Trust, in England after obtaining approval from the Research and Development Office of East Kent Hospitals University NHS Foundation Trust (approval number: 2022/GAP/02). The first cohort comprised patients who received single-marker templated THRs between August and December 2021. The second cohort included those undergoing THRs templated with the KingMark™ system from January to April 2022. Exclusion criteria encompassed patients undergoing THR due to trauma, those requiring revision arthroplasty, individuals with a history of prior hip surgery, cases with developmental abnormalities affecting hip anatomy, and instances of incomplete medical records.

Radiographic technique and templating process

**Figure 1:**
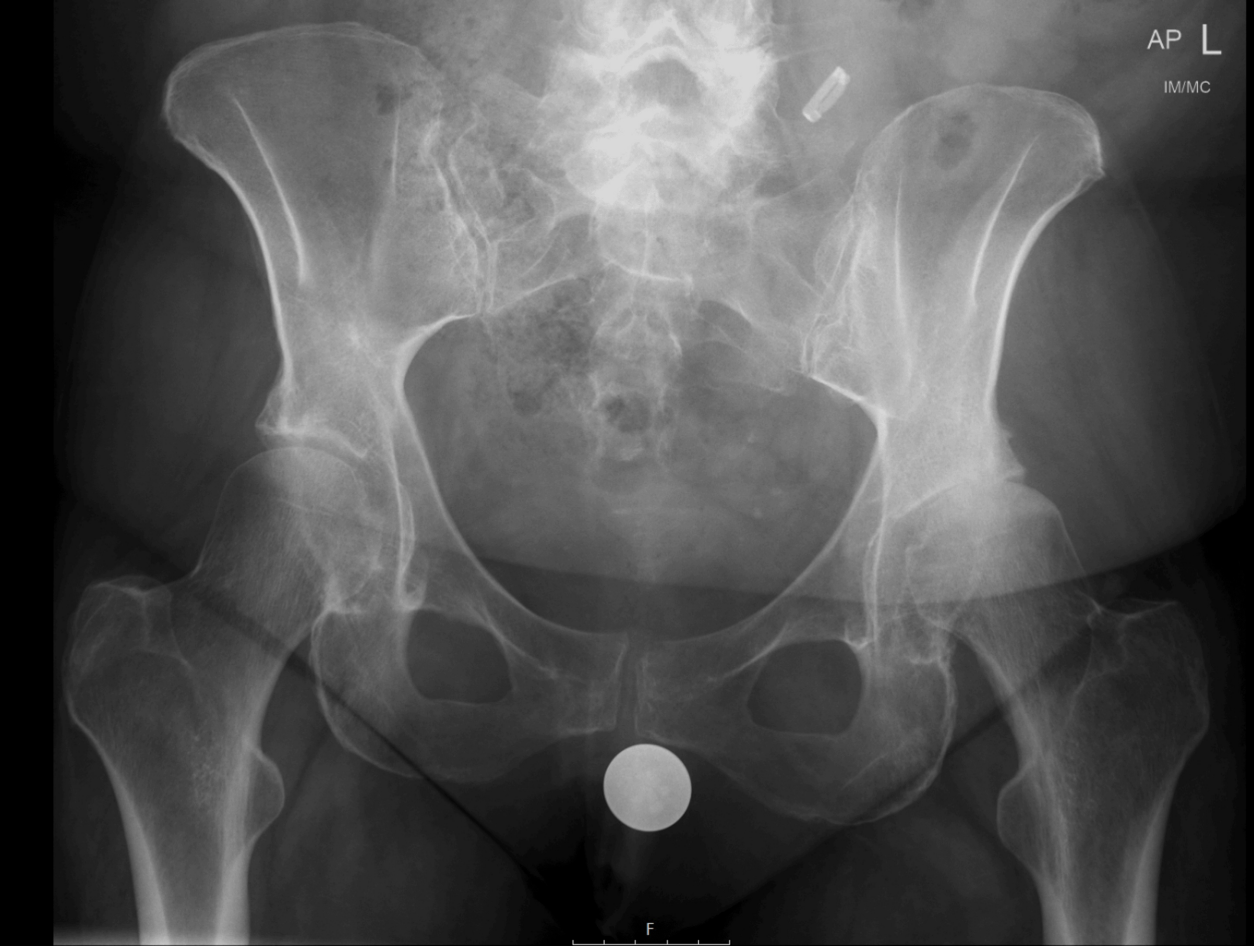
Single-marker templating system

**Figure 2:**
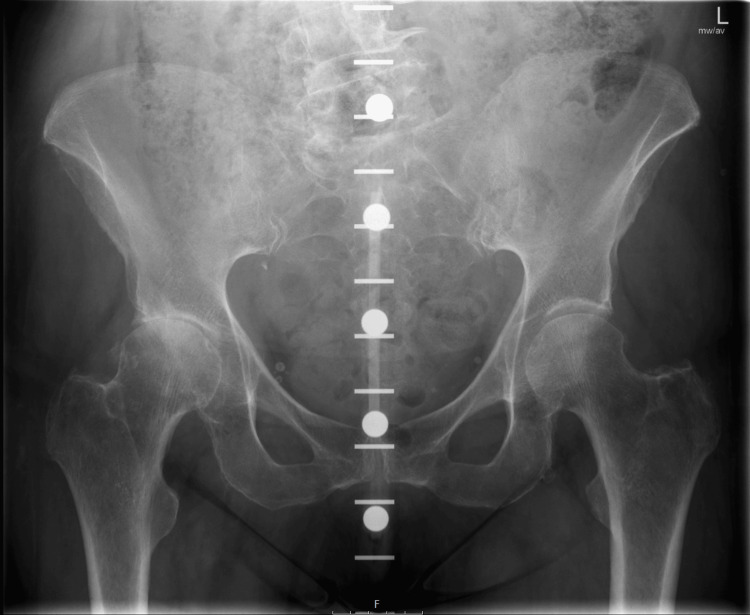
The KingMark™ templating system

Preoperative anteroposterior (AP) pelvic radiographs for all patients were obtained by qualified radiographers following the local radiology protocol and conducted without additional supervision or training. For optimal imaging, patients were positioned supine with the legs extended and slightly apart. Each patient's pelvis was internally rotated by 15 degrees to ensure true AP alignment of the femoral head and neck, minimising distortion and ensuring accurate measurements. In the single-marker technique, a marker (referenced in Figure 1) was positioned at a distance from the X-ray detector that approximated the anticipated location of the femoral head. This positioning of the marker was carefully calibrated according to a standardised protocol to achieve maximum accuracy in distance and alignment. Conversely, the KingMark™ system, illustrated in Figure 2, incorporates a marker worn as a belt around the pelvis, featuring radiopaque markers situated anteriorly and posteriorly. The belt system was adjusted to ensure a snug fit and appropriate positioning of the markers against the skin. Prior to imaging, a thorough calibration was performed to confirm the distance and orientation of the markers relative to the anatomical landmarks.

Both cohorts utilised the same digital templating software, Trauma CAD (Brainlab, Munich), for the templating process. The XMF was rigorously calibrated using a reference scale included within the imaging protocol, ensuring consistency in measurements across all radiographs [11,14]. The software allowed for the precise overlay of templated implants based on the radiographic images, providing a high level of detail and accuracy in the preoperative planning stages (Figure 1 and Figure 2).

Data collection and processing

The radiographs from both cohorts were archived in the Trust's PACS and subsequently transferred to Trauma CAD for preoperative templating. The templated implant data for all cases were saved to the hospital PACS, which remained accessible for this study. Definitive implant information was extracted from physical ledgers in the operating theatre department, which meticulously records all implanted materials. Given that femoral implant size comprises multiple parameters (including stem size and offset length), each configuration was assigned a unique numerical code for expedient data analysis.

Statistical analysis

Statistical analysis was done using Stata Statistical Software: Release 15.1 (November 2017; StataCorp LLC, College Station, Texas, United States). The first set of analyses examined the characteristics of the two groups to examine how comparable they were. Categorical variables were compared between groups using the chi-squared test, whilst the unpaired t-test was used for the continuous outcomes. The next analyses compared the outcome variables between groups. As the outcomes were all found to be approximately normally distributed, the analyses were performed using the unpaired t-test. 

Demographic and clinical characteristics were evaluated utilising descriptive statistics. Continuous variables were expressed as mean±standard deviation, while categorical data were reported as frequencies and percentages. Comparative analyses between the two templating methodologies were performed, and the absolute differences between templated and definitive implant sizes were computed and contrasted across the two cohorts. A p-value of less than 0.05 was deemed statistically significant.

## Results

Fifty patients were recruited into both cohorts with comparable demographics (Table 1). The mean age of the single-marker cohort was slightly higher, at 72.6 years, compared to the KingMark™ at 70.5 years. Female patients comprised the majority of both the single-marker and KingMark™ cohorts, at 64% and 60%, respectively.

**Table 1:** Demographic and procedural characteristics

Variable	Category	Single marker (n=50)	KingMark™ (n=50)
Gender	Female	32 (64%)	30 (60%)
	Male	18 (36%)	20 (40%)
Age	-	72.6±10.4	70.5±9.3
Procedure side	Left	22 (44%)	26 (53%)
	Right	28 (56%)	23 (47%)

The single-marker method correctly predicted definitive femoral implant size in 32% of cases compared to 54% via the KingMark™ method. This was a statistically significant improvement in the rate of correct femoral implant prediction (p=0.04). The mean templated acetabular size for the single-marker cohort was 52.5±4.1, and the mean definitive acetabular cup size was 53.6±3.5. The mean templated acetabular cup size in the KingMark™ cohort was 53.2±4.8, and the mean definitive acetabular cup size was 53.2±4.8. The absolute difference between templated and definitive acetabular implants was 2.3±2.4 and 2.2±2.6, respectively, which was not statistically significant (p=0.84). This is consistent with the rate of accurate acetabular implant prediction for both templating methods (32% for single marker and 30% for KingMark™) with no significant difference (p=0.83) (Table 2 and Table 3).

**Table 2:** Acetabular implant prediction

Cup size difference	Single-marker number (%)	Cumulative	KingMark™ number (%)	Cumulative
0	16 (32%)	32	15 (30%)	30
±1	19 (38%)	70	20 (40%)	70
±2	8 (16%)	86	13 (26%)	96

**Table 3:** Outcome variables ^*^Difference between the KingMark™ and single-marker values with 95% confidence interval ^+^Calculated as implanted size minus templated size ^#^Absolute value of the difference between the implanted and templated sizes

Outcome	Single marker	KingMark™	Difference (95% CI)^*^	P-value
Acetabular cup templated	52.5±4.1	52.0±3.7	-0.5 (-2.1, 1.1)	0.53
Acetabular cup implanted	53.6±3.5	53.2±4.8	-0.5 (-2.1, 1.2)	0.57
Acetabular cup difference^+^	1.1±3.2	1.2±3.2	0.0 (-1.2, 1.3)	0.98
Acetabular cup absolute difference^#^	2.3±2.4	2.2±2.6	-0.1 (-1.1, 0.9)	0.84
Acetabular correct prediction	16 (32%)	15 (30%)	-2 (-20, 16)	0.83
Femur size templated	11.0±2.6	9.9±3.6	-1.1 (-2.3, 0.2)	0.09
Femur size implanted	11.6±2.5	10.2±3.7	-1.4 (-2.7, -0.2)	0.03
Femur size difference^+^	0.6±1.2	0.2±1.0	-0.4 (-0.8, 0.1)	0.11
Femur absolute difference^#^	1.0±0.9	0.6±0.8	-0.4 (-0.7, 0.0)	0.04
Femur correction prediction	16 (32%)	27 (54%)	22 (3, 41)	0.03

## Discussion

Despite the reported increased accuracy of the KingMark™ system, our findings have not shown a statistically significant difference between it and the conventional single marker for predicting the acetabular component. The templated size for the acetabular component correctly predicted the implanted size in 30% of cases in the KingMark™ cohort compared with 32% in the single-marker cohort. However, the KingMark™ system is more accurate in predicting the femoral component.

Warschawski et al. have reported no significant difference between the two templating methods when investigating 126 cases that underwent preoperative templating. Only 49 cases were templated using KingMark™, and the other 79 cases were templated using a sizing ball. The prediction accuracy for the acetabular component was roughly 74% for both systems. It was noted that the femoral stem prediction was superior for the dual-marker system; however, this did not reach statistical significance (p=0.15) [15]. In their series, accurate implant size prediction was defined as an exact match ± one size in contrast to our study, where an accurate prediction was described as an exact match. Conversely, the Al-Ashqar et al. study reported that KingMark™ was superior to conventional single-marker templating at predicting the acetabular component. However, the techniques have equal accuracy in femoral stem prediction after investigating 225 templated patients, 121 by KingMark™ and 104 by single marker [16]. They claimed that the inaccuracy in the prediction of the femoral component stemmed from the malrotation of the femoral head during preoperative radiographs; ideally, the femur should be internally rotated by 15 degrees to measure the actual offset and canal diameter [17].

Girgis et al. reviewed 453 THRs templated with the KingMark™ system and compared them to 189 THRs, which had preoperative templating with a single marker or no marker. In their series, the KingMark™ had predicted an exact match for the acetabular component in 47% of cases; when allowing for a tolerance of ± one size, this improved to 87.4% [18]. This is more accurate than in our series, demonstrating an exact match rate of 30% for KingMark™, increasing to 70% with a tolerance of ± one size. Their reported accuracy of 65.3% for KingMark™'s prediction of the femoral component also exceeds our findings of 54%. Despite this, they found no significant difference when comparing their results to those of their control group, which had virtually the same accuracy for the acetabular (46.7%) and femoral (67.2%) components. They concluded that while templating preoperatively is essential, they could not demonstrate the superiority of one templating method. Notably, most of their comparative cohort used no marker to calibrate magnification.

The purported magnification error for the single-marker sizing ball (4.8-5.95%) is more than four times the quoted error for the KingMark™ double-marker (1.1-1.14%) [10,14]. The correct placement of the single marker at the level of the greater trochanter could reduce this error by 3% [10]. However, lateral marker placement, which aids in estimating the greater trochanters' level, is associated with projection errors [6,19]. In comparison, the KingMark™ method is less technically demanding for radiographers. The hemispherical shape of the acetabular component means it is less affected by radiographic projection and is, therefore, easier to template [16]. Our data supports the superiority of the KingMark™ method of templating for the femoral component and its equivalence in templating the acetabular component. The overall improved accuracy offered by the KingMark™ templating method would result in better preoperative information about implants. This could reduce sub-optimal implant use by ensuring the required component size is available and potentially providing a financial benefit for departments by improving the efficiency of stock inventory.

Limitations

This study has several limitations. First, the sample size is limited, as it was conducted at a single centre with the aim of providing preliminary comparative data between templating methods. While the findings are relevant and applicable given the widespread use of the templating systems studied, broader validation in larger, multicentre studies is warranted. Second, body mass index (BMI) data were not consistently available for all patients, which may affect calibration accuracy. This represents an area for further investigation. Third, the retrospective study design inherently introduces potential biases. To mitigate this, we applied strict inclusion and exclusion criteria and utilised consistent radiographic protocols and data sources. Nevertheless, future prospective studies are necessary to validate and expand upon these results.

## Conclusions

The KingMark™ dual-marker system showed significantly greater accuracy in predicting the size of femoral stems used in THR surgery than the conventional single-marker method. However, there was no significant difference between the two templating methods when predicting acetabular cup size. This indicates that other factors beyond magnification calibration may affect cup size selection during the procedure.

These findings emphasise the need for ongoing refinement of preoperative templating techniques and highlight the importance for surgeons to remain adaptable in their approach, as templating should be viewed as a guide rather than an absolute determinant of implant selection. Future studies should further explore improved templating accuracy's clinical and economic impacts on surgical outcomes and hospital resource utilisation. Additionally, research into the potential benefits of combining advanced imaging technologies with refined calibration methods may further enhance preoperative planning for total hip arthroplasty.
